# Two Cases of Rhabdomyolysis (Haff Disease) After Eating Carp Fish

**DOI:** 10.1177/2324709616663230

**Published:** 2016-08-29

**Authors:** Joey V. Louis, Saw Sein, Claudia Lyon, George Apergis

**Affiliations:** 1 NYU Lutheran Medical Center, Brooklyn, NY, USA

**Keywords:** rhabdomyolysis, foodborne diseases, fishes, heat-stable, Haff disease

## Abstract

Unexplained rhabdomyolysis after eating fish is a rare condition caused by an unidentified toxin. Most of the incidences in the United States have been linked to consuming buffalo fish or crawfish. We present 2 cases of Haff disease in which the patients consumed grass carp as opposed to the usual suspects of buffalo fish or crawfish.

## Introduction

Unexplained rhabdomyolysis within 24 hours after eating cooked fish is a rare condition known as Haff disease and is caused by an unidentified toxin. The first reported case in the United States was in Texas in 1984, with a total of 29 cases of Haff disease reported in the United States from 1984 to 2014.^1^ Most of the incidences in the United States have been linked to consuming buffalo fish, crawfish, or Atlantic salmon.^2^ Carp fish (Cyprinidae family) is similar to buffalo fish (Catostomidae family), but is not the same species.^3^ We present 2 cases of Haff disease in which the patients consumed grass carp as opposed to the usual suspects of buffalo fish or crawfish.

## Case Presentations

### Case 1

A 67-year-old female with a past medical history of hypertension and hyperlipidemia presented to the emergency department 4 hours after eating white fish (grass carp) with generalized body pain and weakness, associated with 3 episodes of nonbilious and nonbloody vomiting the previous night, followed within minutes by back pain that started in her neck and bilateral shoulder region and spread downwards with generalized fatigue and weakness. She stated that her back pain was continuous and stabbing in nature and her muscles felt floppy. She found it difficult to stand and walk. The patient did not have any fingertip or perioral paresthesia, chest pain, shortness of breath, palpitations, dizziness, abdominal pain, or diarrhea. She denied hematuria or any urinary symptoms, vision or speech changes. She had no recent history of a respiratory virus or travel. Her history was significant for hypertension and hypercholesterolemia. She was on the following medications: Lipitor 10 mg (for about 7 years prior), ASA, HCTZ/Losartan, Pepcid, and Simethicone. Lipitor was discontinued on admission. She was not a smoker, alcohol drinker, or drug abuser. She called her daughter who also suffered the same symptoms. The patient reported that both she and her daughter ate the same white fish last night at 9:30 pm; her symptoms started at 2:00 am and were getting worse. This was not their first time eating this type of fish.

Her physical exam on presentation was notable for mild diffuse muscle tenderness and difficulty to stand from weakness, with normal sensations and deep tendon reflexes. Baseline vitals were normal. Baseline significant laboratories were the following: serum creatinine kinase (CK) 19 124 UL, aspartate transaminase (AST) 861 IU/L, and alanine transaminase (ALT) 227 IU/L. She was admitted to intermediate intensive care unit and treated for significant acute rhabdomyolysis. This patient was started on 3 L of 0.9% NS bolus in the emergency department and then maintenance fluid with N/S @ 150 cc/h. She received sodium bicarbonate 8.4% injection, SOLN (sodium bicarbonate 150 MEQ in 5% dextrose 1000 mL at 100 mL/h), and her urinary output, renal function, liver function test, serial CK and myoglobin levels, and serum electrolytes, especially NaHCO_3_ and calcium levels, were monitored accordingly.

**Figure 1.**
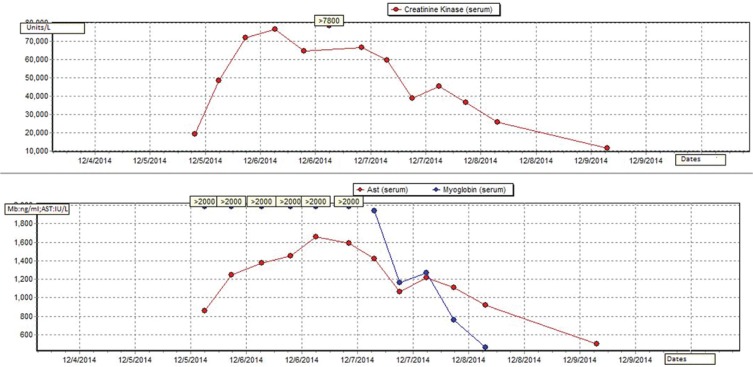
Serial AST, CK, and myoglobin trends in Patient 1.

Her serum CK peaked at 76 380 IU/L, approximately 30 hours after fish exposure (Figure 1). Her transaminase peaked at AST/ALT 1656/576 IU/L and then resolved. Hepatitis serology was negative. Fluid hydration was continued throughout her hospital stay. Her symptoms started to improve on hospital day 2. Over the course of 5 days, CK slowly trended down to 4724 IU/L and she was discharged with instructions to follow-up with her primary medical doctor in 1 to 2 days and check liver function and CK levels. Although age >80 years and statin medications are both risk factors for rhabdomyolysis, it seems more likely that fish consumption was the cause due to her history of low-dose Lipitor without complications and the fact that the other person who consumed this fish became ill simultaneously.

### Case 2

The daughter of the first patient presented to the emergency department at the same time as the mother. The daughter was a healthy 40-year-old female who presented with whole body muscle cramps, weakness, and fatigue associated with bilateral upper and lower extremity numbness and slow movement. She had no gastrointestinal symptoms or pain. Her symptoms progressively worsened, and she started to feel short of breath and experience hematuria. Her baseline laboratories were the following: serum CK >7800 U/L, AST/ALT 587/174 IU/L. She was also admitted for rhabdomyolysis and was treated with the following fluid replacement and monitoring: 0.9% NS bolus followed by 150 mL/h, monitor urinary output, renal function, liver function test, serial CK and myoglobin levels, and check serum electrolytes, especially NaHCO_3_ and calcium levels, replaced as needed.

Her CK peaked at 61 000 IU/L. On hospital day 2, she felt much better and could move her legs, but she complained of chest pain when she took a deep breath. Her renal function remained normal throughout the admission and hepatitis profile was negative. She was discharged to follow-up with her primary care provider to evaluate CK and kidney function.

## Discussion

Rhabdomyolysis is a condition in which there is injury to muscle cells, resulting in breakdown of the cells, and leakage of those contents into the blood stream. This leads to the characteristic dark urine due to the elevated serum CK levels, muscular pain and tenderness, stiffness, weakness, edema, and impaired mobility.^4^ Injured skeletal muscle releases different forms of CK but CKMM is the most prevalent isoform of skeletal muscle and what is measured to diagnose rhabdomyolysis. In rhabdomyolysis of clinical importance, this enzyme level commonly reaches 100 000 IU/L or more.^4^ Acute rhabdomyolysis should be suspected at any CK level above 5000 IU/L, since total CK levels rarely exceed that in cardiomyopathy.^4^

Haff disease is a rare form of rhabdomyolysis that was first reported in the Baltic region in 1924, after the consumption of cooked fresh water fish^1^ (Haff: *German* “Lagoon”). In addition, this report suggested that the warning issued by it did not pertain to carp fish, because the 3 people who developed Haff disease at that time consumed buffalo fish, a bottom feeder similar to carp. In one of the cases attributed to buffalo fish in 1997, the couple in Missouri who got Haff disease had consumed carp in addition to the buffalo fish.^5^ In animal studies, researchers were able to induce Haff disease in mice and cats by feeding them carp from a pond.^6^ This seems to indicate that the fish by itself may not be the source of the toxin.

During investigations of the outbreaks, it was observed that the people who developed the disease had eaten cooked fish, which suggested that the toxin responsible must be heat stable.^5^ The toxin appears to be related to palytoxin, which produces palytoxin poisoning.^7^ Haff disease, however, appears to be related to mostly to freshwater fish consumption, as opposed to marine fish. This characteristic is unlike most of the other seafood-related illnesses^8^ and also separates the Haff disease toxin from the palytoxin found in marine fish. Though it is not confirmed, some toxin candidates have emerged. One of them belongs to water hemlock, a plant called “beaver poison” that grows in wetlands, which produces cicutoxin.^9^ Fish can consume water hemlock, which in turn can release cicutoxin in the person consuming the fish. Cicutoxin is a heat-stable toxin that can produce rhabdomyolysis and may be responsible for the symptoms of Haff disease. Further research is necessary to identify the toxin. Both of our patients had eaten the same type of fish before this incident, indicating that the causative factor is not only the species of fish, but whether the fish has consumed the toxin prior to being cooked.

## Conclusion

Fish consumption should be considered as a possible etiology in patients presenting with rhabdomyolysis. In addition, when considering Haff disease in the absence of buffalo fish or crawfish consumption, clinicians should be aware that different kinds of fish could be related to Haff disease. Geographically, Haff disease has been reported across the United States, including cases in New York in 2011, and it is not endemic to a particular area.^10,11^ Early diagnosis should be accompanied by aggressive fluid resuscitation and bicarbonate treatment to prevent renal failure.^12^
